# Idiopathic Gingival Fibromatosis Rehabilitation: A Case Report with Two-Year Followup

**DOI:** 10.1155/2013/513153

**Published:** 2013-03-27

**Authors:** Mahesh Jayachandran, Shalini Kapoor, Rethi Mahesh

**Affiliations:** ^1^Noorul Islam College of Dental Sciences, Aralammoodu PO, Thiruvananthapuram, Kerala 695123, India; ^2^SGT Dental College, Hospital & Research Institute, Gurgaon, Delhi, India

## Abstract

Gingival enlargements are quite common and may be either inflammatory, noninflammatory, or a combination of both. Gingival hyperplasia is a bizarre condition causing esthetic, functional, psychological, and masticatory disturbances of the oral cavity. Causes of gingival enlargement can be due to plaque accumulation, due to poor oral hygiene, inadequate nutrition, or systemic hormonal stimulation (Bakaeen and Scully, 1998). It can occur as an isolated disease or as part of a syndrome or chromosomal abnormality. A progressive fibrous enlargement of the gingiva is a facet of idiopathic fibrous hyperplasia of the gingiva (Carranza and Hogan, 2002; Gorlin et al., 1976). It is described variously as *fibromatosis gingivae, gingivostomatitis, hereditary gingival fibromatosis, idiopathic fibromatosis, familial elephantiasis,* and * diffuse fibroma*. We present a case of idiopathic gingival fibromatosis with its multidisciplinary approach of management.

## 1. Introduction

Idiopathic gingival fibromatosis is characterized by a slowly progressive, benign enlargement, which affects the marginal gingiva, attached gingiva, and interdental papilla. The fibromatosis may potentially cover the exposed tooth surfaces, causing esthetic and functional problems, and in extreme cases may distort the jaws. Gingival tissues surrounding both the maxillary detention and the mandibular dentition may be affected. The hyperplasic gingiva usually presents a normal color and has a firm consistent with abundant stippling [1]. Buccal and lingual tissues may be involved in both the mandible and the maxilla. This anomaly is classified into two types according to its form—the nodular form and the symmetric form. The localized nodular form is characterized by the presence of multiple enlargements in the gingiva. The symmetric form which is the most common type of this disorder results in uniform enlargement of the gingiva. The degree of enlargement may vary from mild to severe. As a result, the teeth become buried, to varying degrees, beneath the redundant hyperplastic tissues. The gingival tissues are usually pink and nonhemorrhagic and have a firm, fibrotic consistency [1, 2]. Most cases of hereditary gingival fibromatosis appear to be inherited in an autosomal-dominant manner, although autosomal recessive inheritance has also been reported. Autosomal-dominant forms of gingival fibromatosis, which are usually nonsyndromic, have been genetically linked to the chromosomes 2p21-p22 and 5q13-q22 [3–5]. In modern times, a mutation in the Son of sevenless-1 (*SOS-1*) gene has been suggested as a possible cause of isolated (nonsyndromic) gingival fibromatosis [3]. However, no definite linkage has been established. Diffuse gingival enlargement is also found to be associated with syndromes like Cross syndrome, Rutherford syndrome, Ramen syndrome, Zimmerman Laband syndrome, and Juvenile hyaline syndrome [1, 2, 6] (Table 1).

## 2. Case Report

**Figure 1:**
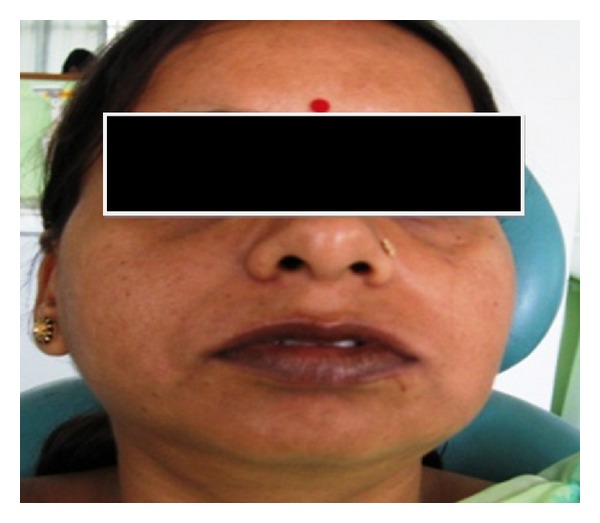
Preoperative photograph with fullness of face.

A 30-year-old woman reported complaining of disfigurement of face due to swelling in gums since seven years, which was causing functional and masticatory difficulty. She presented with a generalized severe gingival overgrowth, involving the maxillary and mandibular arches and covering almost the whole dentition. The patient was also concerned about the progressively increasing space between her upper front teeth and their movement away from their original positions. Extra oral examination (Figure 1) revealed a convex profile with bimaxillary protrusion, incompetent lips, and malocclusion. 

Patient's medical and family history was noncontributory. The patient was not receiving any antiepileptic, antihypertensive, or immunosuppressive medications that could contribute to the gingival enlargement. Past dental history reveals that patient got surgical treatment (gingivectomy) done for the same twice, but the condition had reoccurred.

**Figure 2:**
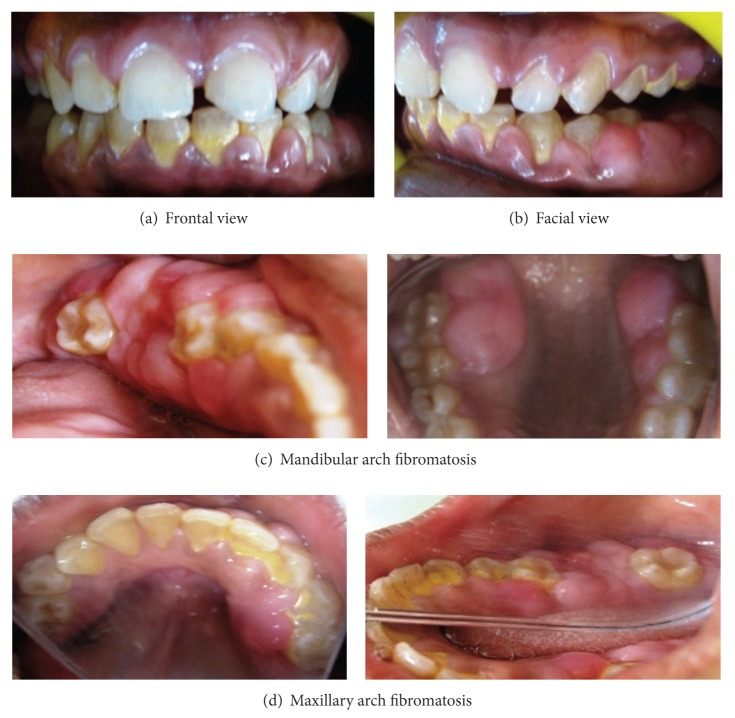
Preoperative intraoral picture of generalized gingival fibromatosis.

Intraoral examination revealed enlargement of the gingiva on both buccal and lingual/palatal sides with pinkish red, fibrous inconsistency and absence of stippling. Gingival enlargement enclosed the major surface of the teeth present except the incisal/occlusal surfaces. Severe diffuse enlargement involving the marginal, interdental, and attached gingiva of both arches, covering almost all the surfaces of the teeth, was found. There was generalized spacing in the dentition with proclined maxillary anteriors (Figure 2).

Clinical examination revealed mobility in all the teeth present and severe pathologic migration, especially of the upper anterior teeth. There were deep pockets present and there was an increase in the intermaxillary rest position. Physical examination of the whole body and blood investigations were advised to eliminate any medical abnormalities.

The radiographic findings corroborated those of the clinical examination and revealed severe generalized alveolar bone loss, which could be attributed to the local factors which must have exaggerated the hyperplastic condition. The peripheral blood results were normal and correlated with an absence of any history of systemic disease. Based on all these findings, a provisional diagnosis of idiopathic gingival enlargement was made.

### 2.1. Treatment

Treatment decided was full mouth undisplaced flap surgery.

#### 2.1.1. Surgical Stage

After routine phase one periodontal therapy a treatment plan was formulated which comprised of quadrant-wise undisplaced flap surgery (the only treatment of choice in this condition as we had to treat the patient's underlying periodontal disease). The treatment procedure was explained to the patient and written consent was obtained. The surgery was planned under local anesthesia containing 2% lignocaine with 1 : 200000 epinephrine. Undisplaced flap surgery was performed to excise desired quantity of soft tissue. The wound was irrigated with betadine and a Coe-Pak (noneugenol, hard, and fast set) was given for seven days. Patient was advised to take analgesics and rinse twice daily with 0.2% chlorhexidine mouthwash. The excised tissue was sent for histopathological evaluation.

#### 2.1.2. Histopathological Report

 The sections revealed moderately dense collagenous connective tissue with collagen bundles arranged in a haphazard manner. Connective tissue was relatively avascular along with scanty inflammatory cell infiltrate showing dense wavy bundles of collagen fibers containing numerous fibrocytes and fibroblasts. The overlying epithelium was hyperplastic with elongated rete ridges. The histopathologic features led to the final diagnosis of idiopathic gingival fibromatosis.

#### 2.1.3. Maintenance Phase

**Figure 3:**
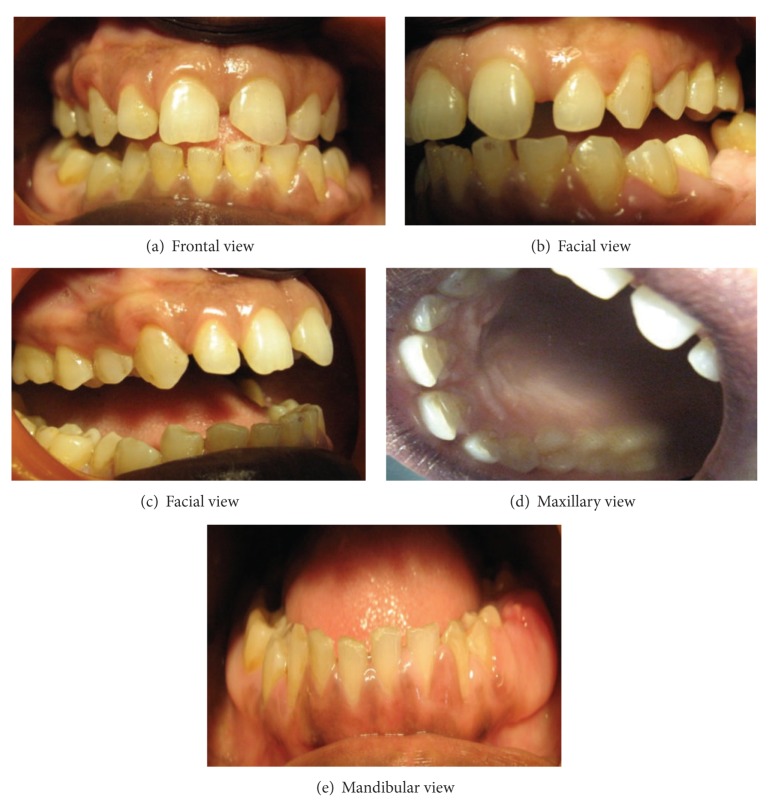
Postoperative intraoral picture of generalized gingival fibromatosis.

The case was followed up for 6 weeks postoperatively and then every 3 months for 2 years. The mobility of the teeth was reduced to physiologic at the end of 3 months. No recurrence was observed within 2 years. Patient is still following the follow-up regime. Mild recurrence in the right maxillary posterior palatal segment was seen after 1 year (Figure 3). 

## 3. Discussion

This paper reports a case of idiopathic gingival fibromatosis. It may be congenital or hereditary. Gingival overgrowth varies from mild enlargement of isolated interdental papillae to segmental or uniform and marked enlargement affecting one or both jaws [7]. In the present case, patient had no history of any systemic disease, hypertrichosis, mental retardation, epilepsy, or medication which could contribute to gingival overgrowth. She also did not give history of pregnancy. General physical examination of the patient revealed no syndromic association which could contribute to gingival overgrowth. The clinical, histopathological features and systemic examination excluded the diagnosis of neoplastic enlargement, hereditary gingival fibromatosis, Wegener's granulomatosis, acanthosis nigricans, and idiopathic variety [5, 8]. 

The precise mechanism of idiopathic gingival fibromatosis is unknown, but it is seen often to confine to the fibroblasts which harbor in the gingivae. The hyperplastic response does not involve the periodontal ligament and occurs peripheral to the alveolar bone within attached gingivae [7]. The continuing recurrence of the enlargement after repeated surgery and a permanent remolding of tissues after extraction of teeth suggest the importance of the presence of teeth and the gingival crevice environment in the pathogenesis of gingival fibromatosis [7]. In our case classically in those sites where extraction has been done there was no gingival overgrowth present.

 There is inconsistency in the literature as to the cellular and molecular mechanisms that lead to gingival fibromatosis. Some authors report an increase in the proliferation of gingival fibroblasts [9], whereas others report slower-than normal growth [10]. Increased collagen synthesis rather than decreased levels of collagenase activity may be involved [9]. The constant increase in the tissue mass can result in delayed eruption and displacement of teeth, arch deformity, spacing, and migration of teeth. The condition is not painful until the tissue enlarges to partially cover the occlusal surface of the molars, thereby getting traumatized during mastication. In the present case, enlargement was not found to cover the occlusal surface, although due to massive gingival enlargement, the patient experienced difficulty in speech and maintaining oral hygiene measures. This could have resulted in the accumulation of materia alba and plaque, which could have further complicated the existing situation.

The surgical treatment of choice is the gingivectomy, which was first advocated for drug-induced gingival enlargement in 1941. As there is, in nearly all circumstances, adequate attached gingiva, there is little fear of creating mucogingival problem with this technique. However, in this case we opted for undisplaced flap procedure to treat the patient's periodontal disease as well as to improve esthetics. Since the literature reports high recurrence rate after surgery present case has been monitored closely for improvement in her periodontal condition for two years with a mild recurrence in the right maxillary posterior palatal segment. Patient was advised to maintain good oral hygiene to minimize the effect of inflammation on fibroblasts.

## 4. Conclusion

Our present case was of nonsyndromic idiopathic gingival enlargement, with its surgical management and followup for a period of two years. Treatment was undisplaced flap surgery, which appreciably improved the patient's aesthetic and masticatory competence as well as her periodontal condition.

## Figures and Tables

**Table 1:** Syndromes associated with gingival fibromatosis [6].

Syndrome	Clinical features	Mode of inheritance
Laband syndrome	Syndactily, nose, and ear abnormalities, hyperplasia of the nails, and terminal phalanges	Dominant
Rutherfurd syndrome	Corneal dystrophy	Dominant
Cross syndrome	Microphthalmia, mental retardation, and pigmentary defects	Recessive
Ramon syndrome	Hypertrichosis, mental retardation, delayed development epilepsy, and cherubism	Recessive
